# A decomposition of Fisher’s information to inform sample size for developing or updating fair and precise clinical prediction models – part 3: continuous outcomes

**DOI:** 10.1186/s41512-026-00228-9

**Published:** 2026-04-13

**Authors:** Rebecca Whittle, Richard D Riley, Lucinda Archer, Gary S Collins, Amardeep Legha, Kym IE Snell, Joie Ensor

**Affiliations:** 1 Department of Applied Health Sciences, School of Health Sciences, College of Medicine and Health, University of Birmingham, Birmingham, UK; 2 National Institute for Health and Care Research (NIHR) Birmingham Biomedical Research Centre, Birmingham, UK; 3 Institute of Data and AI, University of Birmingham, Birmingham, UK

**Keywords:** Clinical prediction models, Sample size, Confidence intervals, Instability, Fisher’s information matrix, Fairness, Continuous outcomes

## Abstract

**Background:**

Clinical prediction models enable healthcare professionals to estimate individual outcomes using patient characteristics. Current sample size guidelines for developing or updating models with continuous outcomes aim to minimise overfitting and ensure accurate estimation of population-level parameters, but do not explicitly address the precision of predictions. This is a critical limitation, as wide confidence intervals around predictions can undermine clinical utility and fairness, particularly if precision varies across subgroups.

**Methods:**

We propose methodology for calculating the sample size required to ensure precise and fair predictions when developing models with continuous outcomes using linear regression. Building on Fisher’s unit information matrix theory, our approach calculates how sample size impacts the epistemic (model-based) uncertainty of predictions and allows researchers to either (i) evaluate whether an existing dataset is sufficiently large, or (ii) determine the sample size needed to target a particular confidence interval width around predictions. The method requires real or synthetic data representing the target population. To assess fairness, the approach can evaluate prediction precision across subgroups. Extensions to prediction intervals are included to additionally address aleatoric uncertainty.

**Results:**

We demonstrate the methodology by examining the sample size required to develop or update a model for predicting Forced Expiratory Volume (FEV) using age, height, and sex. Existing guidance suggests a minimum sample size of 237 participants for this setting. We show this corresponds to an anticipated mean confidence interval width of 0.206 L across all participants in the target population, and that widths may be considerably larger for some individuals. Our new approach calculates a minimum of 694 patients would be needed to ensure all anticipated interval widths were ≤ 0.3. Subgroup analysis showed comparable anticipated precision across sex subgroups. Sensitivity analysis assuming conditional independence among predictors yielded consistent results. For prediction intervals, the magnitude of the residual variance imposes a lower bound on interval width, even with very large samples.

**Conclusions:**

Our methodology provides a practical framework for examining required sample sizes when developing or updating prediction models with continuous outcomes, focusing on achieving precise and equitable predictions. It supports the development of more reliable and fair models, enhancing their clinical applicability and trustworthiness.

## Background

Clinical prediction models are vital tools in personalised medicine, supporting healthcare professionals in making informed decisions about diagnosis, prognosis, and treatment, by estimating an individual’s likely outcome based on their characteristics and clinical information (i.e., ‘predictors’). For instance, a model might predict a baby’s expected birth weight at specific gestational ages using predictors such as maternal age, history of diabetes, and ethnicity [1]. These predictions can help clinicians tailor interventions and monitor patients more effectively.

Accurate prediction models can help improve patient outcomes, optimise resource allocation, and enhance the overall quality of care. However, many existing models produce unreliable predictions, often due to flaws in study design and analysis [2–7]. A common issue is the use of datasets that are too small for model development or updating, which can result in imprecise and poorly calibrated predictions [8, 9]. This, in turn, reduces clinical utility and may lead to flawed decision-making in practice [10]. For this reason, the TRIPOD + AI reporting guideline specifically asks authors of prediction model studies to explain how the sample size was arrived at and justify it [11].

When developing models for predicting a continuous outcome, current guidance on sample size calculations suggests that the minimum sample size should aim to minimise model overfitting and ensure precise estimation of the overall mean value and the residual variance [12].

However, these guidelines do not account for the uncertainty in predictions, which can remain substantial due to model instability, even when the recommended minimum sample size is met [8, 9, 13]. Large uncertainty results in wide confidence intervals around the predicted outcomes, reducing the model’s reliability for clinical use. The acceptable level of uncertainty will depend on the specific context and outcome of interest.

To ensure that a model provides sufficiently precise predictions for decision making, sample size calculations are needed that specifically target the desired level of precision in outcome estimates. This is particularly important in clinical scenarios where decisions informed by predictions carry significant consequences.

A related consideration is the precision of predictions within relevant subgroups, especially those including underserved populations (e.g., ethnicity, socio-economic status) [8]. Ensuring adequate precision across these groups is essential for fairness and equity in model performance.

In this article, we propose methodology for calculating sample sizes that target precise predictions when developing or updating linear regression-based prediction models for continuous outcomes, building on our previous work for binary and time-to-event outcomes [14, 15]. After briefly summarising existing approaches, we extend them to derive closed-form solutions that can be applied either before data collection, or when an existing or pilot dataset is available. These solutions allow researchers to examine how the width of the confidence interval around a predicted outcome value is anticipated to vary with sample size, given the values of a specified set of predictors. The minimum required sample size can also be determined for a target confidence interval width. To facilitate the approach, we describe methods for simulating datasets that resemble those that a researcher plans to collect, based on summary statistics from published studies. This enables practical implementation of our approach via our software module, *pmstabilityss *[16]. We present applied examples to illustrate the methodology, followed by a concluding discussion. The solutions relate to minimising epistemic uncertainty (model-based error), but we extend to also consider prediction intervals (epistemic and aleatoric uncertainty).

## Existing sample size approach for developing a prediction model with a continuous outcome

We have previously derived calculations for the minimum sample size needed to predict continuous outcomes based on targeting the following criteria [12]:

- Criterion (i): a global shrinkage factor $\:\ge\:$ 0.9.
- Criterion (ii): a small absolute difference in $\:{R}_{adj}^{2}$ and $\:{R}_{app}^{2}$.
- Criterion (iii): precise estimation of the residual standard deviation
- Criterion (iv): precise estimation of the mean predicted outcome value (model intercept)

Further details of these sample size calculations are available in previous papers [12, 17]. The approach is implemented in the *pmsampsize* package (available in Stata, R and Python) [18–20], with the user needing to specify the anticipated $\:{R}^{2}$ of the model, the average outcome value, and the standard deviation of outcome values in the population of interest.

For example, in the applied example we consider the development of a linear regression model to estimate Forced Expiratory Volume (FEV) in children based on three predictor parameters: age, sex and height (assuming linear associations for age and height). If we assume that an existing prediction model in the same field has an $\:{R}^{2}$ of 0.77, and that FEV values in the target population have a mean of 2.64 L and standard deviation of 0.87 L, then *pmsampsize* calculates that at least 237 participants are needed to meet criteria (i) to (iv).

## New sample size approach to target precise estimates of continuous outcomes

We now introduce a new method to help researchers assess how the sample size for model development may influence the precision and fairness of outcome estimates. The approach is grounded in linear regression and maximum likelihood theory and is also applicable when extending or updating an existing model, especially when existing or pilot data are available. This is because it requires researchers to input information on anticipated case-mix distributions and model performance. The method provides an initial target sample size that can inform, for example, grant applications or study planning. We emphasise that once data collection and analysis is underway, internal validation remains essential to assess model stability and performance, for example by using learning curves and stability plots [8, 21, 22].

### Step 1: identify the predictors expected to be included in the model

In addition to sample size and model performance (see later), the uncertainty of a prediction for a continuous outcome depends on the predictors included in the model. Therefore, to estimate prediction uncertainty, it is essential to identify the predictors most likely to be included in the model being developed; referred to here as the *core predictors*. The set of core predictors are typically those with well-established predictive value in the relevant clinical context but may also include emerging predictors of interest.

Core predictors can be identified through previously published models, systematic reviews of prognostic factors [23], or in consultation with clinical experts. For example, when predicting systolic blood pressure (SBP), core predictors might include age, sex, BMI, ethnicity, and diabetes status [24–27] .

While additional predictors may be considered during model development, potentially increasing the uncertainty of predictions, the core predictors and their value combinations provide a foundational basis for assessing sample size and the precision of predictions.

### Step 2: identify or create a relevant dataset reflecting the target case-mix distribution

The joint distribution of the predictors (i.e., the case-mix distribution) included in the final prediction model affects the standard errors of the linear regression model’s parameter estimates, thereby influencing the width of the confidence intervals around predictions. Therefore, in Step 2, the joint distribution of the core predictors identified in Step 1 must be specified for the target population for model development. The method for doing this depends on the information available at the time of sample size calculation:

#### Scenario 1: existing dataset or pilot data are available to the model development team

This is a common situation when a team is planning to develop or update a prediction model. It represents the simplest case for calculating sample sizes using the proposed method, as a dataset containing the predictors and their joint distribution is already available, so can be used to define the case-mix distribution for the sample size calculation. In this scenario, the user can proceed directly to Step 3.

#### Scenario 2: no existing data are available

If individual-level data are not currently available to the model development team, data can be simulated to approximate the anticipated joint distribution of the core predictors in the target population.

If a previously published article reports results from both univariable and multivariable linear regression models that include the core predictors of interest for a population similar to the target population, this information can be used to estimate the joint distribution of the core predictors. Specifically, the covariance between each core predictor, $\:x$, and the outcome, $\:y$, can be calculated using the reported regression coefficient, $\:{\beta\:}_{uni}$, from a univariable regression of the outcome on the predictor, using the following formula:

1 $$\:\mathrm{C}\mathrm{o}\mathrm{v}\left(x,y\right)={\beta\:}_{uni}\times\:\mathrm{V}\mathrm{a}\mathrm{r}\left(x\right).$$

This assumes that the variance of the predictor is either reported or can be calculated (e.g., from a reported standard deviation), and that all predictors are continuous. If binary predictors are included (including dummy variables for categorical predictors), their variance can be calculated as $\:p(1-p)$, where $\:p$ is the proportion of individuals with the predictor value equal to 1.

Once the covariances between each predictor and the outcome are obtained, they can be combined with the vector of regression coefficients from a multivariable model, $\:{\boldsymbol{\beta\:}}_{\boldsymbol{m}\boldsymbol{u}\boldsymbol{l}\boldsymbol{t}\boldsymbol{i}}$, to estimate the covariances between the predictors using:

2 $$\:\mathrm{C}\mathrm{o}\mathrm{v}\left(\boldsymbol{X}\right)={\boldsymbol{\beta\:}}_{\boldsymbol{m}\boldsymbol{u}\boldsymbol{l}\boldsymbol{t}\boldsymbol{i}}^{-1}{\left(\mathrm{C}\mathrm{o}\mathrm{v}\left({X}_{i},y\right)\right)}^{{\prime\:}}.$$

The variance-covariance matrix in Eq. (2) can be solved using simultaneous equations and can then be used to simulate a dataset reflecting the joint distribution of the core predictors in the reference population.

If a relevant dataset exists but is not yet available (e.g., access is conditional on funding approval), the data holders may be able to provide a synthetic dataset that mimics the joint distribution of the predictors. For example, the Clinical Practice Research Datalink (CPRD) has previously provided synthetic datasets to support protocol development. Alternatively, data holders may provide the variance-covariance matrix of the predictors, allowing the research team to simulate a dataset that reflects the expected joint distribution, without needing to use approximations.

If it has not been possible to obtain information on the joint distribution of the predictors, either because a new dataset needs to be collected or because data holders were unable to provide the necessary information, researchers may still simulate a dataset using available summary statistics (e.g., means, standard deviations, or proportions for categorical variables). These can often be extracted from published tables of baseline characteristics from studies in the target population. In this case, the predictors are assumed to be conditionally independent, meaning no correlations are specified between them. The impact of this assumption might be explored through sensitivity analyses, for example by simulating datasets under different assumed correlation structures. We consider this in an example later.

### Step 3: derive the Fisher’s unit information matrix

Now assume we have a dataset of size $\:N$ from Step 2, comprising individuals indexed by $\:i=1,\dots\:,N$. As mentioned, this dataset characterises the joint distribution of the core predictors (i.e., to reflect the anticipated target population) and in Step 3 is now used to estimate the corresponding Fisher’s unit information matrix. Note that we distinguish between this dataset of size $\:N$ (which is merely used to define the case-mix distribution) and the sample size $\:n$, which is the sample size of interest for developing or updating the prediction model, to be examined in subsequent steps.

For each individual, let $\:{y}_{i}$ denote the observed value of a continuous outcome, and let

$$\:{\boldsymbol{x}}_{\boldsymbol{i}}={\left(1,{x}_{1i},{x}_{2i},\dots\:,{x}_{pi}\right)}^{\mathrm{T}}$$

denote the vector of observed predictor values for that individual, for the $\:p$ core predictors identified in Step 1. Stacking these vectors yields the $\:N\times\:\left(p+1\right)$ design matrix $\:\boldsymbol{X}$.

For the derivations that follow, we assume a linear regression model of the form

$$\:{y}_{i}={\boldsymbol{x}}_{i}^{\mathrm{T}}\boldsymbol{\beta\:}+{\epsilon}_{i},$$

where $\:\boldsymbol{\beta\:}$ is the vector of regression coefficients and the error terms, $\:{\epsilon}_{i}$, are assumed to be independent and identically distributed as

$$\:{\epsilon}_{i}\sim\mathrm{N}\left(0,{\sigma\:}^{2}\right).$$

Unlike in logistic regression and survival models [28, 29], the precision of a prediction from a linear regression model is not conditional on the actual predicted value itself. However, it still depends on the model’s overall performance (e.g., explained variation), the sample size ($\:n$) used for model fitting, and the individual’s predictor values. Uncertainty in predicted outcome values arises from uncertainty in the estimated regression coefficients and scales with the sample size $\:n$ used for model development (epistemic uncertainty). Aleatoric uncertainty due to residual variability is considered separately when extending the approach to prediction intervals (see Extensions).

Model parameters are estimated using maximum likelihood, which coincides with ordinary least squares under these assumptions. Conditional on the design matrix $\:\boldsymbol{X}$, the variance-covariance matrix of the estimator $\:\widehat{\boldsymbol{\beta\:}}$ for a model developed using a sample size $\:n$ is

$$\:\mathrm{C}\mathrm{o}\mathrm{v}\left(\widehat{\boldsymbol{\beta\:}}\right)=\frac{{\sigma\:}^{2}}{n}{\left(\frac{1}{N}{\boldsymbol{X}}^{\mathrm{T}}\boldsymbol{X}\right)}^{-1}.$$

Recall that $\:N$ is the size of the (synthetic) dataset used to define the anticipated case mix distribution in the target population, whereas $\:n$ is the sample size for the model development data. To disentangle the effect of sample size, $\:n$, from the predictor distribution and residual variance, we express the variance-covariance matrix of the estimated regression coefficients $\:\mathrm{C}\mathrm{o}\mathrm{v}\left(\widehat{\boldsymbol{\beta\:}}\right)$ as:

3 $$\:\mathrm{C}\mathrm{o}\mathrm{v}\left(\widehat{\boldsymbol{\beta\:}}\right)=\frac{1}{n}{\boldsymbol{I}\left(\boldsymbol{\beta\:}\right)}^{-1},$$

where the Fisher’s unit information matrix is defined as:

4 $$\:\boldsymbol{I}\left(\boldsymbol{\beta\:}\right)=\frac{1}{{\sigma\:}^{2}}\frac{{\boldsymbol{X}}^{\mathrm{T}}\boldsymbol{X}}{N}.$$

The Fisher’s information matrix corresponds to the expected information contributed by all observations, whereas the Fisher’s *unit* information matrix represents the expected information contributed by a single observation. This decomposition relies on the assumption that observations are independent and identically distributed, so that the total information is the sum of the information contributions from individual observations. As a result, the Fisher’s information matrix can be expressed as the product of the sample size $\:n$ and the Fisher’s unit information matrix, which provides the novel ‘trick’ that motivates the implementation of our sample size calculation, below.

To use Eq. (3) for sample size calculation, we must specify the anticipated value of $\:\boldsymbol{I}\left(\boldsymbol{\beta\:}\right)$. This depends on the value of $\:{\boldsymbol{X}}^{\mathrm{T}}\boldsymbol{X}$ (derived from the dataset of size *N* in Step 2) and the residual variance $\:{\sigma\:}^{2}$, which must be specified by the researcher. Alternatively, the anticipated $\:{R}^{2}$ of the model can be specified, which is related to the residual variance through the following expression:

5 $$\:{\sigma\:}^{2}=\frac{\left(1-{R}^{2}\right)\sum\:{\left({y}_{i}-\stackrel{-}{y}\right)}^{2}}{N},$$

where $\:\stackrel{-}{y}$ is the mean value of the outcome.

Substituting Eq. (5) into Eq. (4), the Fisher’s unit information matrix can be re-expressed as:

6 $$\:\boldsymbol{I}\left(\boldsymbol{\beta\:}\right)=\frac{{\boldsymbol{X}}^{\mathrm{T}}\boldsymbol{X}}{\left(1-{R}^{2}\right)\sum\:{\left({y}_{i}-\stackrel{-}{y}\right)}^{2}}.$$

Thus, to compute the Fisher’s unit information matrix, we need:

- The $\:{\boldsymbol{X}}^{\mathrm{T}}\boldsymbol{X}$ matrix derived from the data set specified in Step 2 and, either apply Eq. (4) using:
- an assumed value of the residual variance, $\:{\sigma\:}^{2}$ or apply Eq. (6) using:
- an assumed value for $\:{R}^{2}$, and
- the assumed sum of squares of the outcome, $\:\sum\:{\left({y}_{i}-\stackrel{-}{y}\right)}^{2}$.

For the latter, any pilot or existing dataset from Step 3 should also have an outcome variable; however, if not, the outcome variable $\:y$ can be simulated using a known or assumed mean and standard deviation, to estimate $\:\sum\:{\left({y}_{i}-\stackrel{-}{y}\right)}^{2}$. Importantly, the outcome in the simulated data does not need to be conditionally dependent on the predictors, as the Fisher’s unit information matrix is based only on the predictor distribution and residual variance, not on the predictor-outcome relationship.

### Step 4: examine the impact of sample size on the precision of outcome estimates

The final step involves assessing how the size of the (planned or existing) dataset intended for model development or updating may affect the precision of outcome predictions, as reflected in the width of anticipated 95% confidence intervals around predicted values. This is particularly relevant when using an existing dataset for model development or updating, to examine whether this existing dataset is already sufficiently large for use, and, if not, to ascertain how much further data collection is needed to achieve appropriate target sample sizes. The sample size calculations can consider two options:

#### Option A: calculate the anticipated confidence interval widths of predictions for a given sample size

This option applies when a dataset already exists for model development. The prediction ($\:{\mu\:}_{new}$) for a new individual is their expected (mean) outcome value conditional on their predictor values $\:{x}_{new}$, defined using

$$\:{\mu\:}_{new}=\mathrm{E}\left({y}_{new}|{x}_{new}\right)={\boldsymbol{x}}_{new}^{\mathrm{T}}\boldsymbol{\beta\:},$$

which is estimated by

$$\:{\widehat{\mu\:}}_{new}={\boldsymbol{x}}_{new}^{\mathrm{T}}\widehat{\boldsymbol{\beta\:}}\:.$$

The variance of the predicted (expected) outcome value ($\:{\widehat{\mu\:}}_{new}$) for new individuals with the same set of predictor values is given by:

7 $$\:\mathrm{V}\mathrm{a}\mathrm{r}\left({\widehat{\mu\:}}_{new}\right)=\mathrm{V}\mathrm{a}\mathrm{r}\left({\boldsymbol{x}}_{new}\widehat{\boldsymbol{\beta\:}}\right)={\boldsymbol{x}}_{new}\mathrm{V}\mathrm{a}\mathrm{r}\left(\widehat{\boldsymbol{\beta\:}}\right){\boldsymbol{x}}_{new}^{{\prime\:}},$$

where $\:{\boldsymbol{x}}_{new}=(1,{x}_{1,new},{x}_{2,new},\dots\:,{x}_{\mathrm{p},new})$ are the predictor values for the new individual.

Substituting Eq. (3) from Step 3, we get:

8 $$\:\mathrm{V}\mathrm{a}\mathrm{r}\left({\widehat{\mu\:}}_{new}\right)=\frac{1}{n}{\boldsymbol{x}}_{new}{\boldsymbol{I}\left(\boldsymbol{\beta\:}\right)}^{-1}{\boldsymbol{x}}_{new}^{{\prime\:}}.$$

If we had an estimate of the individual’s predicted outcome, we could derive an anticipated confidence interval around this estimate using:

9 $$\:\left[{\widehat{\mu\:}}_{new}\pm\:\left({t}_{a/2,n-p}\sqrt{\mathrm{V}\mathrm{a}\mathrm{r}\left({\widehat{\mu\:}}_{new}\right)}\right)\right]=\left[{\widehat{\mu\:}}_{ne{w}_{L}},{\widehat{\mu\:}}_{ne{w}_{U}}\right],$$

where $\:{t}_{a/2,n-p}$ is the critical value from the $t$-distribution with $\:n-p$ degrees of freedom, $\:n$ is the sample size, $\:p$ is the number of parameters, and L and U denote the lower and upper bounds, respectively.

For large sample sizes (typically *n* > 100, which we would expect when using *pmsampsize* as a starting point), the $\:t$-distribution can be approximated by the standard normal distribution, where $\:{z}_{\alpha\:/2}$ is the critical value from the standard normal distribution.

Although the predicted value $\:{\mu\:}_{new}$ is unknown at the sample size calculation stage, we can use Eq. (8) to compute the width of the confidence interval (CI) as:

10 $$\begin{aligned} \mathrm{CI}\;\mathrm{width}&=2\times t_{\alpha/2,n-p-1}\sqrt{Var\left({\widehat\mu}_{new}\right)}\;\\&=2\times t_{\alpha/2,n-p-1}\;\sqrt{\frac1n\left({\boldsymbol x}_{new}\boldsymbol\;\boldsymbol I\left(\boldsymbol\beta\right)^{-1}\boldsymbol x_{new}^{'}\right)}.\end{aligned}$$

This allows the user to evaluate the anticipated confidence interval widths for individuals in the existing or simulated dataset (from Step 2), using the Fisher’s unit information matrix $\:\boldsymbol{I}$***(β)*** from Step 3 and a specified sample size $\:n$. These widths can then be assessed for clinical acceptability, either overall, or within specific subgroups.

#### Option B: calculate a target sample size for new data collection

When planning a new study involving prospective data collection, researchers may wish to determine the sample size required to achieve a desired level of precision in outcome predictions. Rearranging Eq. (8), the required sample size to achieve a target prediction variance is:

11 $$\:n=\frac{1}{\mathrm{V}\mathrm{a}\mathrm{r}\left({\widehat{\mu\:}}_{new}\right)}{x}_{new}{\boldsymbol{I}\left(\boldsymbol{\beta\:}\right)}^{-1}{x}_{new}^{{\prime\:}}.$$

Alternatively, this can be expressed in terms of a target confidence interval width, assuming a normal distribution:

12 $$\:n=\frac{{\boldsymbol{x}}_{new}{\boldsymbol{I}\left(\boldsymbol{\beta\:}\right)}^{-1}{\boldsymbol{x}}_{new\:}^{{\prime\:}}}{{\left(\frac{\mathrm{C}\mathrm{I}\:\mathrm{w}\mathrm{i}\mathrm{d}\mathrm{t}\mathrm{h}}{2\times\:{z}_{\alpha\:/2}}\right)}^{2}}.\:$$

This calculation can be applied to each individual in the dataset from Step 2 to determine the required sample size for their specific predictor profile $\:{\boldsymbol{x}}_{new}$, given a maximum acceptable confidence interval width for the outcome of interest.

## Applied example

To illustrate the proposed methodology, we consider the development of a prediction model for estimating Forced Expiratory Volume (FEV) in children and adolescents. The goal is to determine the sample size required to ensure precise predictions of FEV from a linear regression model, with age, sex, and height as predictors. Moreover, an existing dataset containing lung function measurements from 654 individuals is freely available [30], and so the calculations aim to examine the anticipated precision of predictions from this dataset (in advance of analysis), to help ascertain (e.g. for a grant funding application) whether it is fit for purpose.

We explore two practical scenarios: one in which researchers have access to the existing dataset and can use it directly to inform the sample size calculations (Scenario 1), and another in which access to the raw data is not available, but relevant summary statistics can be obtained from published sources or data custodians (Scenario 2). These scenarios reflect common situations in clinical research and allow us to demonstrate how the proposed methodology can be flexibly applied depending on the level of data access. In both cases, our aim is to determine the minimum sample size required to achieve a target level of precision in predictions, as quantified by the average width of the confidence intervals, and to ascertain whether the existing dataset of 654 participants is likely appropriate. We now walk through the four steps outlined above.

### Step 1: identify the predictors expected to be included in the model

For this example, we assume that age, height, and sex are the core predictors of interest for predicting FEV, and that they are linearly associated with the outcome. These variables are well-established in the literature as key predictors of lung function in children [31].

### Step 2: identify or create a relevant dataset reflecting the target case-mix distribution

#### Scenario 1: existing dataset or pilot data are available to the model development team

We use the existing dataset of 654 children aged 3–19 years, collected in East Boston, United States, during the 1970’s [30]. This dataset includes information on FEV (in litres) and our three core predictors (age, height, and sex). It is openly available at https://hbiostat.org/data/. Since the joint distribution of the three core predictors can be directly observed in this dataset, no simulation to generate data is required, and we can proceed to Step 3.

#### Scenario 2: no existing data are available

Now suppose we do not have access to the individual-level data. Instead, we assume that the data owners are not willing to share their data until grant funding is obtained, but they do provide the summary statistics and regression coefficients presented in Tables 1 and 2.

**Table 1:** Summary information for core predictors and the outcome available from previous publication

Characteristic	*N* = 654
Age (years); *Mean (SD)*	9.9 (2.95)
Height (inches); *Mean (SD)*	61.1 (5.7)
Sex (male); *N (%)*	336 (51.38%)
FEV (litres); *Mean (SD)*	2.64 (0.87)

**Table 2:** Model coefficients for the core predictors from a previous publication

	Univariable Coefficient (95% CI)	Multivariable Coefficient (95% CI)
Age (years)	0.222 (0.207, 0.237)	0.061 (0.044, 0.079)
Height (inches)	0.132 (0.126, 0.138)	0.105 (0.095, 0.114)
Sex (male)	0.361 (0.231, 0.492)	0.161 (0.096, 0.226)

Using the standard deviations (to calculate the variance) from Table 1 and the univariable coefficients from Table 2, we calculate the covariances between each predictor and the outcome using Eq. (1). We then use these covariances, along with the multivariable coefficients, to solve the system of equations derived from Eq. (2) to estimate the variance-covariance matrix of the predictors:

$$\:\mathrm{C}\mathrm{o}\mathrm{v}\left(\boldsymbol{X}\right)=\left(\begin{array}{ccc}\mathrm{V}\mathrm{a}\mathrm{r}\left({x}_{age}\right)&\:\mathrm{C}\mathrm{o}\mathrm{v}\left({x}_{age},{x}_{height}\right)&\:\mathrm{C}\mathrm{o}\mathrm{v}\left({x}_{age},{x}_{sex}\right)\\\:\mathrm{C}\mathrm{o}\mathrm{v}\left({{x}_{age},x}_{height}\right)&\:\mathrm{V}\mathrm{a}\mathrm{r}\left({x}_{height}\right)&\:\mathrm{C}\mathrm{o}\mathrm{v}({x}_{height},\:{x}_{sex})\\\:\mathrm{C}\mathrm{o}\mathrm{v}({x}_{age},{x}_{sex})&\:\mathrm{C}\mathrm{o}\mathrm{v}({x}_{sex},{x}_{height})&\:\mathrm{V}\mathrm{a}\mathrm{r}\left({x}_{sex}\right)\end{array}\right).$$

This results in the following system of equations:

$$\begin{aligned} \begin{array}{c}\:{\beta\:}_{age}Var\left(x_{age}\right)+{\beta\:}_{height}Cov\left({x_{age},x}_{height}\right)+{\beta\:}_{sex}Cov\left(x_{age},x_{sex}\right)=Cov\left(x_{age},y\right)\\\:{\beta\:}_{age}Cov\left({x_{age},x}_{height}\right)+{\beta\:}_{height}Var\left(x_{height}\right)+{\beta\:}_{sex}Cov(x_{sex},x_{height})=Cov\left(x_{height},y\right)\\\:{\beta\:}_{age}Cov\left(x_{age},x_{sex}\right)+{\beta\:}_{height}Cov(x_{height},\:x_{sex})+{\beta\:}_{sex}Var\left(x_{sex}\right)=Cov\left(x_{sex},y\right)\end{array}\end{aligned}$$

These equations can be solved using software such as R (using *solve()*) or Python (using *sym.Eq*). Once the variance-covariance matrix is obtained, we assume multivariate normality to simulate a dataset using the *mvrnorm()* function in R, generating *n* = 1,000,000 observations (R code to replicate the example can be found at https://github.com/RebeccaWhittle).

Since sex is a binary variable, we convert the simulated continuous values into binary by sorting and assigning the top 48.62% as female (sex = 0) and the remainder as male (sex = 1), based on the observed proportion in Table 1.

We also simulate the outcome variable (FEV) from a normal distribution with the reported mean and standard deviation. Importantly, this outcome does not need to be conditionally dependent on the predictors for the purpose of estimating the Fisher’s unit information matrix in Step 3.

### Step 3: derive the Fisher’s unit information matrix

With a dataset now available for each scenario considered in Step 2 (either the original dataset of 654 children or a large dataset simulated in Step 2), we proceed to compute the Fisher’s unit information matrix, $\:\boldsymbol{I}\left(\boldsymbol{\beta\:}\right)$, for each using Eq. (6).

For illustrative purposes, we assume an anticipated $\:{R}^{2}$ value of 0.77, chosen to reflect a plausible level of explained variance for a model including age, height, and sex in this clinical context. This value was based on previous prediction models for FEV and previous analyses using subsets of the same data, which is akin to when using a pilot study for new data collection.

The matrix $\:{\boldsymbol{X}}^{\mathrm{T}}\boldsymbol{X}$ is calculated from the design matrix of the core predictors (age, height, sex) from the dataset in Step 2, and similarly the sum of the squares of the outcome $\:\sum\:{\left({y}_{i}-\stackrel{-}{y}\right)}^{2}$ is computed using the observed or simulated FEV values from Step 2.

### Step 4: examine the impact of sample size on the precision of outcome estimates

Finally, both options A and B of Step 4 were applied to examine and determine the sample size required to target appropriately narrow confidence intervals based on the Fisher’s unit information matrices from Step 3.

#### Option A: calculate the anticipated confidence interval widths of predictions for a given sample size

Option A can be used to calculate the anticipated confidence intervals for predicted outcomes in individuals within either the real or simulated dataset from Step 2, conditional on a user-specified sample size. This requires the Fisher’s unit information matrix derived in Step 3, which is then applied in Eq. (10) alongside each individual’s predictor values and the specified sample size to compute the width of the anticipated confidence intervals.

In Scenario 1 (when using the existing dataset), we first calculated the anticipated confidence interval widths for the fixed sample size of *n* = 654, corresponding to the number of observations in the available dataset. This yielded a mean interval width of 0.124, with a range of 0.089 to 0.309. In Scenario 2 (using the simulated dataset), the same sample size of *n* = 654 produced an identical mean width of 0.124, though with a slightly wider range of interval widths (0.089 to 0.352).

**Fig. 1:**
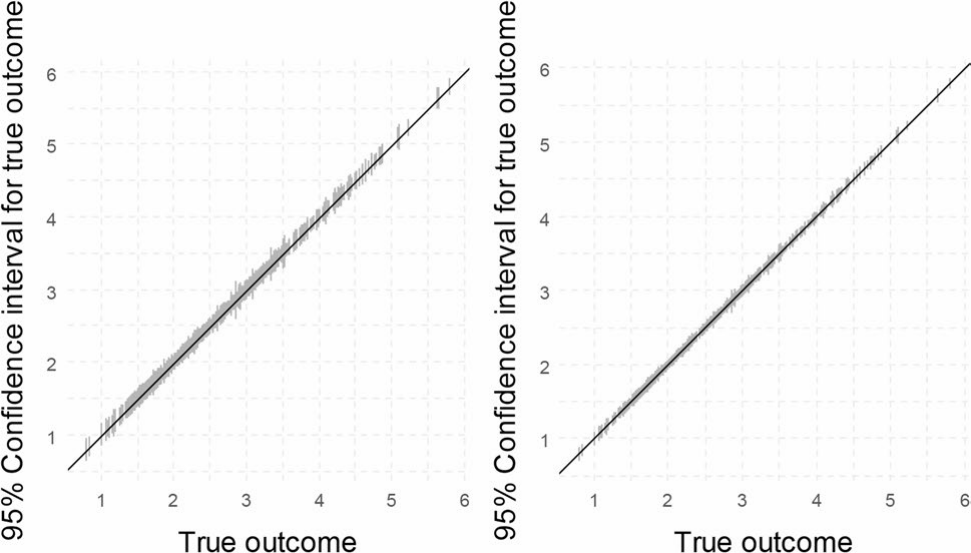
Expected confidence interval widths for predicted values of FEV in the existing dataset when the sample size used for model development is *N* = 237 (left) and *N* = 654 (right)

We also evaluated the confidence interval widths for a smaller sample size of *n* = 237, which corresponds to the minimum required sample size calculated using the Riley et al. criteria described above [12, 18, 19]. As expected, the average confidence intervals were wider, by approximately 0.08 units compared to the larger dataset, and again were quite similar for the two scenarios (Scenario 1: 0.206 (0.148 to 0.513); Scenario 2: 0.207 (0.148 to 0.583)). These results are visualised in Fig. 1, which compares the distribution of anticipated confidence interval widths for individuals in the existing dataset, for both sample sizes. While the increase in confidence interval width at the lower sample size is consistent with theoretical expectations, the absolute difference in this example is small, reflecting the relatively narrow confidence intervals in both sample sizes.

#### Option B: calculate a target sample size for new data collection

We now apply Option B to calculate the minimum sample size required to ensure that *all* predictions have confidence intervals no wider than 0.3 units, which was considered a reasonably acceptable width for this particular clinical scenario. This is achieved by applying Eq. (12) to each individual in the dataset.

Using the existing dataset (Scenario 1), the calculations indicate that a minimum sample size of 694 participants would be required to ensure that all predicted outcomes have confidence interval widths ≤ 0.3. When using the simulated dataset (Scenario 2), the estimated minimum sample size increases slightly to 733 participants, reflecting minor differences in the simulated and real data.

**Fig. 2:**
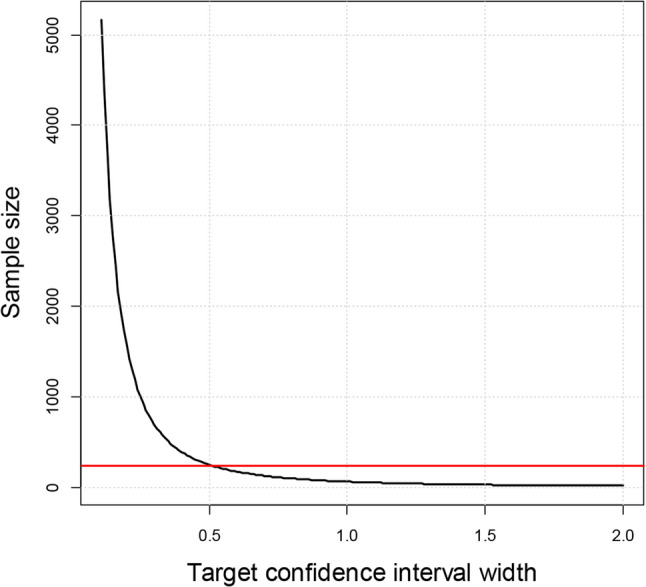
Minimum sample size required to ensure confidence interval widths for all predictions are below a specified threshold in the FEV example. The red line indicates the sample size of 237 (as calculated using *pmsampsize*)

To further illustrate the relationship between sample size and prediction precision, we plotted the minimum sample size required to ensure that all anticipated confidence intervals were at least as narrow as the target width specified on the x-axis (see Fig. 2). The plot shows that at *n* = 237, the minimum recommended by *pmsampsize*, all anticipated confidence interval widths are below 0.51 units, but many are not below 0.3. This highlights the added value of our approach in tailoring sample size calculations to target the precision of predictions.

## Extensions

### Evaluating the confidence interval widths within subgroups

To demonstrate how prediction precision may vary across subgroups, we conducted a stratified analysis by sex. In this context, we focus on one specific aspect of fairness: the consistency of prediction precision across groups. While fairness in clinical prediction models encompasses broader concerns, including biases in data and unequal outcomes, our analysis specifically examines whether one subgroup consistently experiences wider confidence intervals. Such differences in precision may indicate that the model is less reliable for that group, potentially influencing clinical decision making and contributing to inequitable access to healthcare resources.

Using the existing dataset (*n* = 654), we calculated the confidence interval widths separately for male and female participants. This allowed us to evaluate whether the sample size was sufficient to ensure comparable levels of anticipated precision across both groups. The mean confidence interval width anticipated for females was 0.122 (range: 0.091 to 0.309), and for males, it was 0.125 (range: 0.089 to 0.236). These findings suggest that the model will be expected to achieve a comparable level of precision in predictions across sexes, with only minimal differences in confidence interval widths, which is reassuring in terms of fairness of predictions for males and females.

### Assuming predictors are conditionally independent

One of the most significant practical challenges in applying the proposed methodology is the need to specify the joint distribution of the core predictors. This information is essential for calculating the Fisher’s unit information matrix and, in turn, the uncertainty associated with outcome predictions. However, in many real-world scenarios, researchers may only have access to marginal summary statistics (e.g., means, standard deviations, or proportions), without information on the correlations between predictors.

To assess the potential impact of this limitation, we conducted a sensitivity analysis in which we simulated a dataset under the assumption that the core predictors (age, height, and sex) were conditionally independent. This allowed us to evaluate how much the assumption of independence might affect the resulting confidence interval widths.

For a sample size of 654, under the assumption of independent predictors, the confidence intervals had a mean width of 0.124, with a range from 0.088 to 0.351. For a smaller sample size of 237, the mean width increased to 0.206, with a range from 0.148 to 0.583.

These results were highly consistent with those obtained using the true joint distribution of the predictors, suggesting that assuming conditional independence may be a reasonable approximation when full joint distribution information is unavailable. This finding provides reassurance that the methodology can still be applied in settings where only limited information is available, though we recommend that researchers conduct similar sensitivity analyses to assess robustness in their specific context.

### Prediction intervals

So far, our calculations have considered widths of confidence intervals about the expected (mean) value of a prediction given a new individual’s set of predictor values. As the focus is on the uncertainty of the mean value, the confidence interval width only acknowledges model-based error, also known as epistemic uncertainty. However, researchers may also be interested in the *total* uncertainty of a prediction for a new individual, which should acknowledge not only epistemic uncertainty but also aleatoric uncertainty. The latter reflects irreducible variability in the outcome that cannot be explained by the set of (candidate) predictors included in the model, and leads to a prediction interval which will be wider than a confidence interval.

Formally, a prediction interval provides a range within which we expect an individual’s future observed outcome value to fall, accounting for both model uncertainty and residual error. Unlike confidence intervals around *expected* (mean) outcome values, prediction intervals for individual outcome values do not tend to zero even with infinite sample size, because the residual variance remains.

A prediction interval for a new individual’s actual outcome value ($\:{\widehat{y}}_{new})$ is approximately calculated as

13 $$\:{\widehat{y}}_{new}\pm\:\left({t}_{a/2,n-p-1}\sqrt{\mathrm{V}\mathrm{a}\mathrm{r}\left({\widehat{y}}_{new}\right)+{\sigma\:}^{2}}\right),$$

where $\:\mathrm{V}\mathrm{a}\mathrm{r}\left({\widehat{y}}_{new}\right)$ is calculated as in Eq. (8), $\:{\sigma\:}^{2}$ is the residual variance, estimated using Eq. (5), and $\:{t}_{a/2,n-p-1}$ is the critical value from the t-distribution with $\:n-p-1$ degrees of freedom where $\:n$ is the sample size and $\:p$ is the number of predictor parameters (excluding the intercept). For large sample sizes (typically *n* > 100, which we would expect when using *pmsampsize* as a starting point), the $\:t$-distribution can be approximated by the standard normal distribution, yielding:

14 $$\:{\widehat{y}}_{new}\pm\:\left({z}_{\alpha\:/2}\sqrt{\mathrm{V}\mathrm{a}\mathrm{r}\left({\widehat{y}}_{new}\right)+{\sigma\:}^{2}}\right).$$

This expression can be rearranged to estimate the required sample size to achieve a target prediction interval (PI) width:

15 $$\:n=\frac{{\boldsymbol{x}}_{new}{\boldsymbol{I}\left(\boldsymbol{\beta\:}\right)}^{-1}{\boldsymbol{x}}_{new\:}^{{\prime\:}}}{{\left(\frac{\mathrm{P}\mathrm{I}\:\mathrm{w}\mathrm{i}\mathrm{d}\mathrm{t}\mathrm{h}}{2\times\:{z}_{\alpha\:/2}}\right)}^{2}-{\sigma\:}^{2}}.$$

where $\:{z}_{\alpha\:/2}$ is the critical value from the standard normal distribution.

However, this formula has a critical constraint: the denominator must be positive. That is,

$$\:{\left(\frac{\mathrm{P}\mathrm{I}\:\mathrm{w}\mathrm{i}\mathrm{d}\mathrm{t}\mathrm{h}}{2\times\:{z}_{\alpha\:/2}}\right)}^{2}>{\sigma\:}^{2}.$$

This highlights a key property of prediction intervals: the minimum achievable prediction interval width is bounded by the residual variance. In other words, no matter how large the sample size is, even approaching infinity, the aleatoric uncertainty cannot be eliminated. As a result, the prediction interval cannot be made arbitrarily narrow. This underscores the importance of considering the magnitude of residual variance when planning the development of a prediction model.

To illustrate, we return to the applied example. Using the existing dataset (Scenario 1), the residual variance was calculated to be 0.173, and so across all individuals the mean 95% prediction interval width was estimated as 1.634, with a range from 1.631 to 1.658 for a sample size of *n* = 654. For a smaller sample size of *n* = 237, the mean width was 1.642, ranging from 1.635 to 1.708. However, even with *n* = 100,000,000 (i.e., eradicating the epistemic uncertainty), the mean prediction interval width is only marginally reduced to 1.629. This result highlights that when aleatoric uncertainty (i.e., residual variance) is present, increasing the sample size can have only a limited impact on the width of prediction intervals as the uncertainty due to residual variance will always remain. For this reason, generally we recommend to focus sample size calculations on the target confidence interval width.

## Discussion

The reliability of a prediction model is intrinsically linked to the sample size used in its development [10]. While larger sample sizes generally lead to more stable and accurate models, datasets that meet current sample size recommendations, such as those ensuring small optimism in predictor effect estimates, minimal difference between apparent and adjusted $\:{R}^{2}$, and precise estimation of the residual standard deviation and model intercept (as outlined by Riley at [12]), can still yield substantial uncertainty in predictions [8, 9]. In this article, we have extended existing guidance by proposing calculations to determine the minimum sample size required to target precise predictions for models with continuous outcomes. Our approach builds on previous work for binary [14] and time-to-event outcomes [15], and introduces a framework for calculating sample sizes based on target width of the confidence or prediction intervals for individuals in the target population. We provide methods applicable both when individual-level data are available and when only summary statistics can be accessed. As our method assumes unbiased and well-calibrated predictions, we recommend using it in conjunction with existing tools such as *pmsampsize* to ensure that criteria to minimise overfitting and that aim for well-calibrated predictions at the population level are met [12].

In addition to model development, the proposed methodology is also applicable to model updating. Researchers can use information from a previously published model and an existing dataset to assess the expected uncertainty in updated predictions, or to determine the sample size required to achieve a desired level of precision in the updated model.

While this article focuses on model development or complete refitting during updating, the underlying framework may also be relevant for certain model updating settings in which one or more parameters are re-estimated. However, the performance and appropriateness of such applications would require dedicated methodological investigation, which is beyond the current scope of this current work.

Researchers can use this framework in two ways: (i) to evaluate whether an existing dataset is sufficiently large to yield clinically acceptable prediction precision, or (ii) to calculate the required sample size for new data collection based on a target level of prediction precision. Importantly, our approach also supports fairness considerations by enabling researchers to assess and target prediction precision within specific subgroups, such as those defined by age, sex, ethnicity, or socioeconomic status, thereby working towards promoting parity in model performance. Ensuring fairness in clinical prediction models is increasingly recognised as a critical component of responsible model development [11]. A model that performs well on average may still yield systematically less precise or less accurate predictions for certain subgroups, potentially exacerbating existing health disparities. By applying the sample size calculations separately within each subgroup, researchers can evaluate whether the available data are sufficient to ensure acceptable confidence interval widths across all relevant populations. This is particularly important when subgroups are underrepresented in the dataset or when predictor-outcome relationships differ meaningfully between groups. Such assessments may reveal disparities in prediction precision across subgroups.

When this occurs, there is no single optimal response in such situations, and appropriate actions will depend on the clinical or healthcare context, the intended use of the model, and the feasibility of further data collection. Potential responses include collecting additional data to improve precision in under-represented subgroups, restricting the scope of model use to populations for which acceptable precision can be achieved, simplifying the model or revisiting the choice of predictors, or transparently reporting subgroup-specific uncertainty to inform cautious interpretation [2]. Importantly, the proposed methodology enables these issues to be identified prospectively, supporting more informed decisions about model development, updating, and deployment.

However, assessing the consistency of confidence intervals across subgroups is only one aspect of fairness, and even with this approach, it does not guarantee that the resulting models will be fully fair or equitable.

Our method is grounded in maximum likelihood theory for linear regression and uses the Fisher’s unit information matrix to quantify epistemic (reducible model-based) uncertainty [32] via confidence intervals around the mean (expected) predicted value for a new individual. The method focuses on quantifying and minimising this epistemic uncertainty, which arises from estimating model parameters using finite data. This is captured through the Fisher’s unit information matrix and reflects the variability in predictions due to uncertainty in the estimated regression coefficients. By increasing the sample size, we aim to reduce this component of uncertainty and make the model as stable and reliable as possible.

While the primary focus of this article is on epistemic uncertainty, we also included an extension to consider prediction intervals, which incorporate both epistemic and aleatoric uncertainty, the irreducible variability in the outcome that cannot be explained by the model. This total uncertainty sets a lower bound on the achievable precision of predictions, regardless of sample size. In practice, even if model-based uncertainty is minimised, prediction intervals may remain wide as the residual variance is large.

This highlights a fundamental consideration when planning the development or updating of a prediction model: although our method to estimate the sample size required ensures that the model is well-estimated and stable, it cannot overcome limitations imposed by high residual variability. Therefore, researchers must interpret sample size recommendations in the context of the total expected uncertainty in predictions. If the irreducible error is substantial, even a perfectly estimated model may yield predictions that are too imprecise to support clinical decision-making. In such cases, efforts to improve prediction accuracy may need to focus on identifying additional or more informative predictors, rather than simply increasing the sample size, or in some instances, reconsidering whether developing the model is worthwhile at all.

While the derivations are presented specifically for linear regression models estimated using maximum likelihood, this framework may serve as a conceptual reference point for other modelling approaches that yield approximately linear functions of predictor effects or asymptotically multivariate normal parameter estimates. Nonetheless, extensions to other (e.g., machine learning) approaches do require further methodological development.

When using the proposed sample size calculations, researchers must consider several practical factors that influence how the methodology is applied in real-world settings. These include defining what constitutes an acceptable level of uncertainty in predictions, selecting appropriate core predictors for the sample size calculation, and understanding how these choices affect the resulting estimates.

A key practical consideration is defining what constitutes an acceptable confidence interval width. This width will vary depending on the clinical context, the outcome being predicted (and the units of measurement), and the consequences of decision-making based on the model. We recommend that researchers engage with clinicians, patients, and other stakeholders to establish context-specific confidence interval widths for acceptable uncertainty.

Another important consideration is the choice of core predictors used in the sample size calculation. If additional predictors are included during model development, particularly those with low prevalence or weak associations, this may increase the uncertainty of predictions. Sensitivity analyses should be conducted to explore the impact of different predictor sets and assumptions about their joint distributions.

The proposed methodology assumes that predictor and outcome data are complete, or that missing data have been appropriately handled prior to applying the sample size calculations. The approach does not explicitly account for additional uncertainty arising from missing data in predictors or outcomes, which would depend on the extent and mechanism of missingness, and the method used to address it. Extending the framework to formally incorporate uncertainty due to missing data represents an important area for future methodological research. We anticipate that missing predictor data will lead to a larger required sample size, but this needs to be formally evaluated, and the closed form solutions modified accordingly. This is beyond the scope of the present work, but a conservative approach would be to define the sample size as the number of patients with complete predictor data.

One potential barrier to implementation is the need to specify the joint distribution of predictors if researchers do not have access to an existing dataset. However, as demonstrated in our applied example, assuming conditional independence among predictors had minimal impact on the resulting sample size estimates. We also provided pragmatic strategies for approximating joint distributions using published regression coefficients and summary statistics.

In practice, existing or pilot data are often the most straightforward means of characterising the joint distribution of predictors for sample size calculation, which is required to quantify anticipated prediction uncertainty as a function of sample size. However, the validity of the resulting calculations depends on how representative the available data are of the target population. If the existing data are small or not representative, the estimated joint predictor distribution may be imprecise, which could affect the estimated required sample size. Sensitivity analyses using alternative assumptions or simulated predictor distributions may therefore be valuable in practice.

More generally, any assumptions made during sample size calculation may be imperfect, and researchers must rely on using the best information available at the time. In the model development setting, even if the target sample size for model development is attained, internal validation and assessments of model instability remain crucial [8, 9, 33]. In situations when data are prospectively collected, sequential sample size methods may be used to dynamically monitor model development instability as data accrue [21, 22], helping determine when sufficient participants have been recruited and to mitigate the impact of any inaccurate assumptions made at the design stage.

When using simulated data, we observed that the maximum required sample size could vary substantially depending on the random seed and the number of simulated observations. The variability was driven by a small number of extreme predictor profiles, which can arise naturally in large, simulated datasets. To mitigate this, we excluded the most extreme 0.001% of simulated individuals (e.g., the top 10 out of 1 million observations), which led to more stable and consistent estimates that closely matched those obtained in the real dataset. This highlights the importance of applying pragmatic thresholds or sensitivity checks when using simulation-based approaches in general for sample size calculations.

In conclusion, we have proposed a novel method for determining the minimum required sample size to develop or update a clinical prediction model with a continuous outcome, with the aim of targeting precise predictions and to inform fairness checks. This approach allows researchers to assess the impact of sample size on prediction uncertainty and to calculate the required sample size to meet a specified level of precision. By supporting both fairness and precision, our method contributes to the development of more reliable and equitable clinical prediction models.

## Data Availability

The dataset analysed during the current study is openly available at [https://hbiostat.org/data/]. R code to replicate the example can be found at [https://github.com/RebeccaWhittle ].
